# *Melissa officinalis* Protects against Doxorubicin-Induced Cardiotoxicity in Rats and Potentiates Its Anticancer Activity on MCF-7 Cells

**DOI:** 10.1371/journal.pone.0167049

**Published:** 2016-11-23

**Authors:** Alaaeldin Ahmed Hamza, Mahguob Mohamed Ahmed, Hanan Mohamed Elwey, Amr Amin

**Affiliations:** 1 Hormone Evaluation Department, National Organization for Drug Control and Research (NODCAR), Giza, Egypt; 2 Molecular Drug Evaluation Department, NODCAR, Giza, Egypt; 3 Analytical Chemistry Department, NODCAR, Giza, Egypt; 4 Biology Department, UAE University, Al-Ain, UAE; 5 Zoology Department, Cairo University, Giza, Egypt; University of South Alabama Mitchell Cancer Institute, UNITED STATES

## Abstract

Cardiotoxicity is a limiting factor of doxorubicin (DOX)-based anticancer therapy. Due to its beneficial effects, we investigated whether standardized extract of *Melissa officinalis* (MO) can attenuate doxorubicin-induced cardiotoxicity and can potentiate the efficacy of DOX against human breast cancer cells. MO was administered orally to male albino rats once daily for 10 consecutive days at doses of 250, 500 and 750 mg/kg b.wt. DOX (15 mg/kg b.wt. i.p.) was administered on the 8^th^ day. MO protected against DOX-induced leakage of cardiac enzymes and histopathological changes. MO ameliorated DOX-induced oxidative stress as evidenced by decreasing lipid peroxidation, protein oxidation and total oxidant capacity depletion and by increasing antioxidant capacity. Additionally, MO pretreatment inhibited inflammatory responses to DOX by decreasing the expressions of nuclear factor kappa-B, tumor necrosis factor-alpha and cyclooxygenase-2 and the activity of myeloperoxidase. MO ameliorated DOX-induced apoptotic tissue damage in heart of rats. *In vitro* study showed that MO augmented the anticancer efficacy of DOX in human breast cancer cells (MCF-7) and potentiated oxidative damage and apoptosis. Thus, combination of DOX and MO may prove future cancer treatment protocols safer and more efficient.

## Introduction

Doxorubicin (DOX), an anthracycline antibiotic, is regularly used to treat various malignancies, including solid tumors, lymphoma and leukemia. Its association with severe forms of cardiomyopathy and/or congestive heart failure in cancer patients greatly limits DOX application [1–2]. Cellular damage induced by DOX is mediated by the oxidative damage of cardiomyocytes, plasma membranes and the consequent death of those cells by apoptosis [2–3]. Synthetic agents (such as antioxidants and metal chelators) have been investigated with some degree of success [3–4]. While some of those agents such as vitamin E failed to inhibit DOX cardiotoxicity [5] others, such as deferasirox (an iron chelator) increased its toxicity [6]. Cardiotoxicity associated with DOX treatment has been successfully prevented by different medicinal plants [7–9]. Thus, it is well justified to explore more plant derived-natural compounds that prevent the cardiotoxicity of DOX and enhance its chemotherapeutic efficacy.

Among these, *Melissa officinalis* L (MO), Lemon Balm, (Lamiaceae family) is one of the most used medicinal plants in Europe and the Mediterranean region. Normally, herbal tea of MO is used for its aromatic, digestive and antispasmodic properties and to reduce gastrointestinal disorders and sleep disturbance [10–11]. Moreover, MO has been reported to show potent anti-tumor effects in a variety of human cancer cell lines [12–14] and to induce apoptosis in colon carcinoma cells through formation of reactive oxygen species (ROS) [1]. Caffeic acid, protocatechuic acid, rosmarinic acid, ferulic acid, and syringic acid have been reported as the most abundant phenolic compounds in MO [15]. Other phenolic compounds have also been characterized from this plant including triterpene acids and terpenes (ursolic and oleanolic acids and luteolin) [10–11].

The present study was carried out to investigate the protective effect of MO against DOX-induced cardiotoxicity in rats and to elucidate its antitumor effect alone or in combination with DOX on breast cancer cell line (MCF-7). The interest to use the estrogen receptor-positive MCF-7 cell line as a model to evaluate the DOX/MO combinatory anticancer effect stemmed from the evidence that although DOX is among the most active chemotherapeutic drugs for the treatment of breast cancer; it has several limitations particularly in estrogen dependent breast cancer [16]. MCF-7 cell line, thus, serves as an excellent *in vitro* model for studying the mechanisms of chemo resistance as it relates to susceptibility to apoptosis [17]. Markers of oxidative stress, inflammation and apoptosis were also assessed to unravel possible mechanism/s of action of MO.

## Materials and Methods

### Chemicals

All chemicals were of analytical grade and chemicals required for sensitive biochemical assays were obtained from Sigma Chemical Co., St. Louis, MO, USA. Radioimmunoprecipitation assay (RIPA) buffer with protease inhibitors (sc-24948) was purchased from Santa Cruz Biotechnology (Santa Cruz, CA, USA). Poly vinylidene difluoride (PVDF) membrane and blocking reagent were obtained from Roche Diagnostics GmbH (Mannheim, Germany). The primary antibodies used in the western blotting stage were obtained from BioVision (Milpitas, CA, USA) whereas secondary antibody was obtained from Santa Cruz Biotechnology (Santa Cruz, CA, USA). RNA easy Mini Kit (Qiagen, Valencia, CA, USA). TM first strand kit for cDNA synthesis and SYBR Green Real-time PCR Master Mix were bought from Applied Biosystems (Foster City, CA, USA). DOX (Adriblastina, 50 mg) was purchased from Pharmacia Italia S.P.A., Italy.

### Preparation of plant extract

Dried aerial parts (leaves and stems) of MO, grown in May in Syria, where the average temperature is 20°C and average relative humidity is 60%, were purchased from local (Cairo) herbal store. The plant material was authenticated by Dr. Nael M. Fawzi, The Flora and Taxonomy Department, Agricultural Research Center, Giza, Egypt. Plant was stored in light-protected glass bottles at 4°C until the extraction step. Air-dried and ground aerial parts of MO (1000g) were extracted in 70% (v/v) ethanol (2000 mL) by maceration for 48 h at 4°C. The resulting compound was then filter-dried under reduced pressure in a rotary evaporator at 40°C. This crude extract was weighed, dissolved in water for animal study and kept at−20°C for further analysis. The yield of the MO was 12.5 g per 100 g of used plants.

### Ethics statement

Animals were cared for in accordance with the standard guidelines (Canadian Council on Animal Care 1993). The protocol was approved by the Ethics Committee of Animal Care and Use at National Organization for Drug Control and Research, Giza (Approval No 181, 1-7-2015).

### Experimental animals

Adult male Wistar albino rats (Forty eight) were obtained from the animal house of the National Organization for Drug Control and Research (NODCAR). They were maintained on standard pellet diet and tap water *ad libitum* and were kept in polycarbonate clean cages under a 12 hrs. light/dark cycle and room temperature 22–24°C. Rats were acclimatized for two week prior to experimental use.

### Treatment regime

Rats were randomly divided into six groups consisting of eight animals in each group (n = 8) and were subjected to the following treatments: The first group was the control group and received 5ml / kg distilled water through oral gavage for 10 days and injected with single dose of saline (5ml /kg b.wt.) after 7 days of water administration. The second group was the MO group and received 750 mg/kg b.wt. of MO for 10 days. The third group was the DOX-treated group and received a daily dose of distilled water (5ml / kg b.wt.) for 10 days followed by a single intraperitoneal (i.p.) injection of DOX (15 mg/kg) on the 8^th^ day. This DOX dose was selected as it has been used previously to induce acute cardiotoxicity in male albino rats [18]; [19]. Doses of MO were selected based on previously reported pharmacological properties of this plant [20].

DOX solution was freshly prepared in a saline solution. A sample of 1 g MO extract was suspended in 10 ml distilled water. Groups four, five and six received 250, 500 and 750 mg/kg of MO respectively orally and daily for 10 days followed by a single i.p. injection of DOX (15 mg/kg b.wt.) on the 8^th^ day 1 hr after MO treatment. Twenty- four hours after the last MO or vehicle solution administration, blood and heart tissues were collected from all groups and stored at -20°C for further processing.

### Sample preparation

Blood was collected from the retro-orbital plexus and the serum was immediately separated by centrifugation in a refrigerated centrifuge (4°C) at 3000 r.p.m. for 20 minutes. Rats were euthanized by cervical dislocation under diethyl ether anesthesia. The hearts were removed and weighed to calculate the heart to the body weight ratio. Hearts were sliced frontally into two halves (each half includes parts of all chambers of the heart) and for histopathological examination, cardiac tissue samples were immediately fixed in 10% buffered formalin. For biochemical determination, cardiac tissue samples from the other half were homogenized in ice-cold KCl (150 mM). The ratio of tissue weight to homogenization buffer was 1:10. Then, suitable dilutions from that were prepared to determine the levels of oxidative stress biomarkers.

### Biochemical assays and histopathology

#### Cardiotoxicity indices

Aspartate aminotransferase (AST), creatine kinase (CK) and creatine kinase-MB (CK-MB) activities were estimated in serum samples using Randox (Randox Laboratories Ltd., Country Antrim, United Kingdom) and Stanbio (Stanbio laboratory, Boerne, TX, USA) reagent kits and following their instruction manual.

#### Histopathological examination

Pieces of hearts were fixed in 10% neutral phosphate-buffered formalin and hydrated tissue sections, 3μm in thickness, stained with Hematoxylin and Eosin for the histological examinations. The sections were examined under an Olympus DX41 light microscope (Olympus CX31, Honduras St., London, United Kingdom). The sections were graded for average severity of disorganization of normal myfibrillar patterns, focal necrosis, degenerations and inflammations as follows: 0, no change; 1, mild; 2, moderate; and 3, [9] as following: 1, 0–10% of total myocardium; 2, 10–30 total myocardium; 3, more than 30% total myocardium (Table 1).

**Table 1:** Effect of MO treatments on severity of histopathologic lesions in DOX-treated rats.

Groups	Disorganization	Focal necrosis	Degeneration	Inflammation
Control	0.00 ± 0.00	0.00 ± 0.00	0.00 ± 0.00	0.00 ± 0.00
DOX	3.00 ± 0.00^a^	1.83 ± 0.17^a^	2.17 ± 0.31^a^	2.00 ± 0.26^a^
DOX+MO(LD)	1.33 ± 0.21^a^	0.67 ± 0.21^a^	0.67 ± 0.21^a^	1.00 ± 0.36^a^
DOX+MO(MD)	1.00 ± 0.00^a^	0.33 ± 0.21^a^	0.33 ± 0.21^a^	0.67 ± 0.21^a^
DOX+MO(HD)	0.50 ± 0.22^a^	0.17 ± 0.17^a^	0.00 ± 0.00^a^	0.50 ± 0.22^a^

Severity of injury is expressed as mean ± SEM of three scores for seven animals in each group. a P<0.001 vs. DOX group. Grade 1 = 1: 10% mild. Grade 2 = 10–30% moderate. Grade 3 > 30% severe,

^a^ P<0.001 vs. control.

#### Oxidative stress biomarkers

The total oxidant content (TOC) of serum samples was determined as previously described [21]. All the following was assessed in homogenates of heart tissues. Lipid peroxidation was determined by estimating the level of malondialdehyde (MDA) as previously described [22], which is based on its reaction with N-methyl-2-phenylindol to form a blue complex with absorption maximum at 586 nm. P. Carbonyl contents were determined as previously described [23]. Catalase (CAT) activity was determined by measuring the exponential disappearance of H_2_O_2_ at 240 nm and expressed in units/mg of protein as described previously [24]. Superoxide dismutase (SOD) activity was determined according to the method described by Nandi et al [25]. Myeloperoxidase (MPO) activity in cardiac homogenate was determined as previously described [26]. The total protein content of heart was determined according to the Lowry method as modified by [27]. Absorbance was recorded using a PerkinElmer, Lambda 25 UV/VIS spectrophotometer in all measurements.

### Real-time polymerase chain reaction

Total RNA was isolated from 50 mg of heart tissues using RNA Mini Kit (Qiagen, Valencia, CA, USA) according to manufacturer's instruction and further analyzed for quantity and quality with Beckman dual spectrophotometer (USA) at 260 and 280 nm. RNA (5 μg) was then reversed transcribed using revert aid TM first strand cDNA synthesis kit (Ferments life science, Fort Collins, CO, USA). For real time polymerase chain reaction (real time-PCR), the cDNA (5 μL) was subsequently amplified with the Syber Green PCR Master Kit (Applied Biosystems, Foster City, CA, USA) in a 48-well plate using the Step One instrument (Applied Biosystems, Foster City, California, USA). The PCR primers used were designed with Gene Runner Software (Hastings Software Inc., Hastings, NY, USA) from RNA sequences in GenBank and were represented as follows: Forward 5/-GGCGTCCTTCTTGGTTCTGA-3/and Reverse 5/ GGGGACAGCGACACCTTTTA-3/ for NFκB, Forward5′/ACACTCTATCACTGGCATCC-3′/ and Reverse: 5′-GAAGGGACACCCTTTCACAT-3/ for COX-2, Forward 5/-CCAGACCCTCACACTCAGATCA-3′/ and Reverse 5/-TCCGCTTGGTGGTTTGCTA-3′/ for TNF-a, Forward: 5 /-TGTTGTCCCTGTATGCCTCT-3′/ and Reverse 5′-TAATGTCACGCACGATTTCC-3 / for β-actin. PCR samples were denatured at 95°C for 5 min followed 35 cycles that were performed at 95°C for 30 s, 56°C for 30 s, and 68°C for 30 s. Expression of the house- keeping gene β-Actin served as reference gene and values were normalized to the quantity of β-actin. The value of the cycle threshold was used to perform calculations by using the ABI Prism 7500 sequence detection system software as described previously [28]. All signals were expressed relatively to the average values for the control group, which was set to 1. An aliquot of the real time-PCR products (5μl) was separated by electrophoresis on a 1.5% agarose gel containing ethidium bromide and visualized under UV light by a UVP gel imaging system (UVP Co., USA).

### Western blotting analysis

Heart tissue samples (50 mg) were homogenized in cold in RIPA supplemented with inhibitors for proteases and phosphatases and protein concentrations were determined using the established Bradford dye-binding method (Bio-Rad, Hercules, CA, USA). For direct immunoblotting, aliquots of lysate were mixed with loading buffer containing 2-mercaptoethanol and maintained at 100°C for 10 min before loading on 10% SDS-PAGE. Following SDS-PAGE separation, proteins were transferred to PVDF membrane. Membranes were blocked in TBST containing 5% (w/v) non-fat milk and dried for 1 hr at room temperature. Membrane strips were incubated with primary antibodies (diluted 1:1000 for Bax, total caspase-3 and β-actin) overnight at 4°C. The primary antibodies used in this study (BioVision) were diluted at a ratio of 1/200, whereas the secondary antibodies were diluted at a ratio of 1/2000 (Santa Cruz Biotechnology, USA). Following extensive washing, membrane strips were incubated with anti-rabbit IgG (1:5000; Cell Signaling Technology Inc., MA, USA) conjugated to horseradish peroxidase for 1 hr. Protein bands were detected by a standard enhanced chemiluminescence method and densitometry measurements were made using Image J software (Image J; National Institute of Health, Bethesda, USA). The densities of target protein bands were normalized to the corresponding density of β-actin band. All signals were expressed relatively to the average values for the control group, which was set to 1.

### Cytotoxicity activity

Cytotoxic effects of MO, DOX and their combinations were studied against the human breast cancer cell line MCF-7 (ATCC, USA). The cell line was maintained in complete tissue culture medium (Dulbecco’s Modified Eagle’s Medium) with 10% Fetal Bovine Serum and 2 mM-Glutamine, along with antibiotics (about 100 International Unit/mL of penicillin, 100 μg/mL of streptomycin) with the pH adjusted to 7.2. The cytotoxicity was determined by 3-[4,5-dim- ethylthiazol-2-yl]-2,5 diphenyl tetrazolium bromide (MTT assay) [29]. The cells (2X 104/well) were seeded in a 96 well microplate and cultured in the presence of 0, 12.5, 25, 50, 75, and 100 μg/mL of the MO, DOX, and, and MO and DOX, and then were incubated for 24 hrs. The cells were treated with 50 μL MTT reagents (2 mg/mL) and incubated for 3 hrs at 37°C to obtain purple-colored formazan. The color was dissolved in 200 μL of DMSO and measured by an ELISA microplate reader (BioTek Instruments, Winooski, VT) at 570 nm. Cell viability (in percentages, %) was showed as ratio of absorbance (A570 nm) in treated cells relative to absorbance in control cells treated with DMSO (A570 nm). The effects for each compound are expressed by IC_50_ (e.g., the lowest concentration where the effect is inhibited by 50%) and by the magnitude of maximal effect. The IC_50_ values were calculated from dose response curves by computer program GraphPad Prism.

### Quantification of apoptosis

MCF-7 cells were treated with IC_50_ of MO and DOX for 48 hrs and ApoStrand^™^ ELISA apoptosis detection kit (Enzo Life Sciences, BML-AK120, Plymouth Meeting, PA, USA) was used to detect apoptosis in cells according to the manufacturer’s protocol. The ApoStrand^™^ ELISA is based on the sensitivity of DNA in apoptotic cells to formamide denaturation and the detection of the denatured DNA with an antibody to single-stranded DNA.

### Determinations of pro-apoptotic protein levels and intracellular redox state in MCF-7

MCF-7 cells (~2 x10^5^/well) were seeded in 100 mm culture dishes and cultured for 24 hrs. Cells were treated with IC_50_ of MO and DOX for 48 hrs. For combination treatment, cells were treated with equitoxic ratio (half of IC_50_ of each agent) for 48 hrs. After incubation, cells were washed twice with ice-cold PBS, scraped, pelleted and lysed in RIPA buffer (89900, Pierce) supplemented with protease/ phosphatase inhibitor cocktail (1861281, Pierce). After incubation for 30 min on ice, cell lysates were centrifuged at 14,000 rpm for 20 min at 4°C lysates was used for determinations pro-apoptotic proteins and intracellular redox stat. Bax, Cytochrome C and P53 protein expressions were analysis using ELISA Kits (Sun Red, Biotechnology Company, China) according to the manufacturer's instruction. Intracellular redox state in breast cancer cells were evaluated by measuring MDA and nitrite oxide (NO) and glutathione (GSH) levels according to methods described by Arrigo et al [30].

### Antioxidant properties of MO

Total antioxidant properties of MO were estimated by the ferric reducing antioxidant power (FRAP), 1,1-diphenyl-2-picrylhydrazyl (DPPH·), 2, 2-azino-bis (3-ethylbenzothiazoline-6-sulfonate) (ABTS·+). The FRAP assay was determined according to the method of Benzie [31] and DPPH and ABTS methods were determined as previously described [32]. IC_50_ value (mg/mL) is the concentration of extract that causes ABTS·+ or DPPH· radical scavenged by 50%. Also in ABTS, FRAP and DPPH assay, the calibration curve of ascorbic acid was established, the antioxidant capacity of the MO was then expressed as mmol ascorbic acid equivalent/g dry extract.

### Phytochemical analysis

Total phenolic and total flavonoid contents were determined according to the reported procedure described previously [33].

HPLC analyses of phenolic acids were performed according to the method of Barros et al [34] with an Agilent 1200 LC system consisting of degasser, quaternary pump (G1311A), auto sampler (G1329A), column heater (G1316A) and diode array detector (DAD) (G1315C). A reversed phase C18 (4.6 mm × 150 mm × 5 μm) column (Waters Corporation, Milford, MA, USA) was used. The mobile phase used in this study was a gradient of two solvents: solvent A (acetic acid-water, 1:99, v/v) and B (acetonitrile). The gradient profile was as follows from 10% to 15% B; 0–2.5 min, 15% to 25% B; 2.5–5 min, 25% to 35% B; 5–10 min, 50% B isocratic; 10–15 min. The flow rate was 1 mL/min and injection volume was 20 μL. The phenolic compounds present in the samples were characterized according to their UV and retention times, and comparison with authentic standards when available. The detection was carried out at 254, 280, 320 and 360 nm. The HPLC chromatograms monitored at 254 nm revealed more peaks than those at 280 and 320 nm. The wavelength of 254 nm was therefore selected for additional chemical analyses of investigated phenolic acids. HPLC analysis of triterpene acids were performed according to Herodež *et al*. [10]. For quantitative purposes, 2.5–100 μg/mL of different standards were injected and a calibration curve was obtained at 254 nm.

### Statistical analysis

Results of experiments are expressed as means ± S.E.M. SPSS (version 20) statistical program (SPSS Inc., Chicago, IL, USA) was utilized to undergo a one-way analysis of variance (ANOVA) test followed by the Dunnett's test in order to evaluate differences between control and treated groups, and P < 0.05 was considered significant.

## Results

### Cardio protective effect of MO against DOX-induced cardiac damage

#### Effects on cardiac injury marker in serum

**Fig 1:**
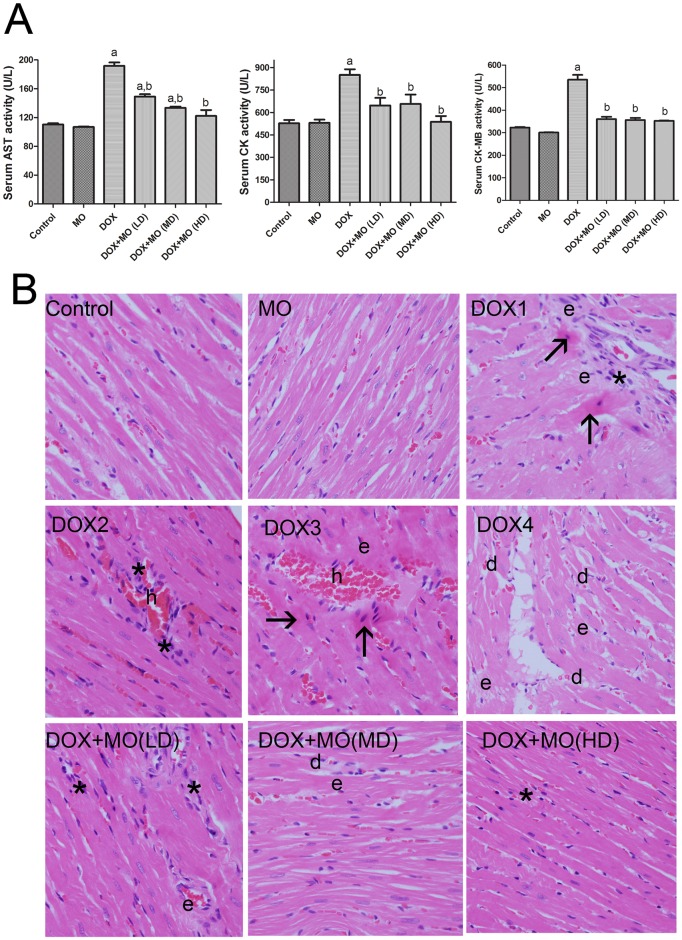
**(A)** Cardio protective effect of MO against DOX-induced cardiac damage, (A) serum cardiac injury markers: AST, CK and CK-MB activities in serum. Data are represented mean ± S.E.M. of seven independent rats of each group. a P<0.05 vs. control; b P<0.05 vs. DOX. (B) Histopathological changes. **(B)** Histopathological changes. Light micrographs of heart sections from control rats showing normal heart architecture, heart sections from DOX groups (DOX1-DOX4) showing focal necrosis with eosinophilic cytoplasm and pyknotic nuclei (arrows) and degeneration of myocardial fibers (d), hemorrhage (h) with inflammatory cell infiltration () and edema (e), In the DOX + LD of MO group, less focal necrosis of muscle fiber and inflammatory cell infiltration (*) with mild edema (e) were noted on cardiac tissue sections. DOX and MD of MO groups showed less degree of degeneration (d), edema (e) and inflammation (*). Animals treated with DOX and HD of MO showed better-preserved appearance of myocardial fibers with mild degree of (e). X 200.

Activities of serum markers, AST and CK, indicating myocardial injury, were significantly elevated in DOX-intoxicated group compared with control. The pretreatment with different doses of MO significantly attenuated the increase in AST, CK and CK-MB levels in DOX-treated group. The decrease in AST, CK and CK-MB activities were dose-dependent while the best protective effect was observed at a high dose of MO (Fig 1A).

#### Histopathological changes

Histopathological analysis of cardiac tissue was undergone to elucidate DOX-induced cardiotoxicity (Fig 1B). Control and MO-only treated groups showed normal myocardium architecture. DOX-treated group showed extensive damage of the cardiac tissue. The lesions were characterized by multiple necrotic foci of myocardial fibers with cytoplasmic vacuole formation and induced eosinophilia, marked edema and accumulation of inflammatory cells. Despite, the persistence of some necrotic foci, edema and inflammation in the cardiac tissues of intoxicated rats treated with lower and moderate dose of MO, pretreatment with high dose of MO showed a well-preserved appearance of myocardial fibers with slight degree of edema.

#### General appearance, mortality, body weight gain and heart /body weight ratio

Rats were observed daily throughout the experiment for pain and distress by monitoring changes in mobility, food/water intake, and body weight. Animals looked healthy except the group treated with DOX alone where rats looked sick and weak and their fur became scruffy. Ascites was present in the DOX administered rats. As shown in Table 2, DOX did not cause significant decrease in heart weight/body weight (HW/BW) ratio however in DOX-treated rats, the body weight gain of rats was significantly decreased in all groups of rats treated with DOX alone or in combination with MO when compared to control group. No significant changes in the BW gain or (HW/BW) ratio were observed in rats treated with MO alone. Animals from the DOX-treated groups at necropsy showed an evident cardiac softness and accumulation of serous fluid in pericardial pleural and peritoneal cavities. Throughout the course of this study, only two mortalities were reported in DOX-treated and DOX+MO (LD)-treated groups.

**Table 2:** Comparison of mortality, body weight (BW) and heart weight/body weight (HW/BW) among experimental groups.

Groups	Mortality	BW gain (g)	HW/BW (x 1000)
Control	0/8	29.57± 1.97	3.06 ± 0.05
MO	0/8	29.93 ± 1.67	3.07 ± 0.04
DOX	1/8	-5.23 ± 1.43^a^	2.89 ± 0.09
DOX+MO (LD)	1/8	-3.34 ± 3.34^a^	3.08 ± 0.12
DOX+MO (MD)	0/8	-2.53 ± 3.53^a^	3.10 ± 0.05
DOX+MO (HD)	0/8	-2.14 ± 1.67^a^	3.10 ± 0.07

Data are represented as mean ± S.E.M. of seven independent rats of each group.

^a^ P<0.001 vs. control.

#### Effect of MO on oxidative stress biomarkers

**Fig 2:**
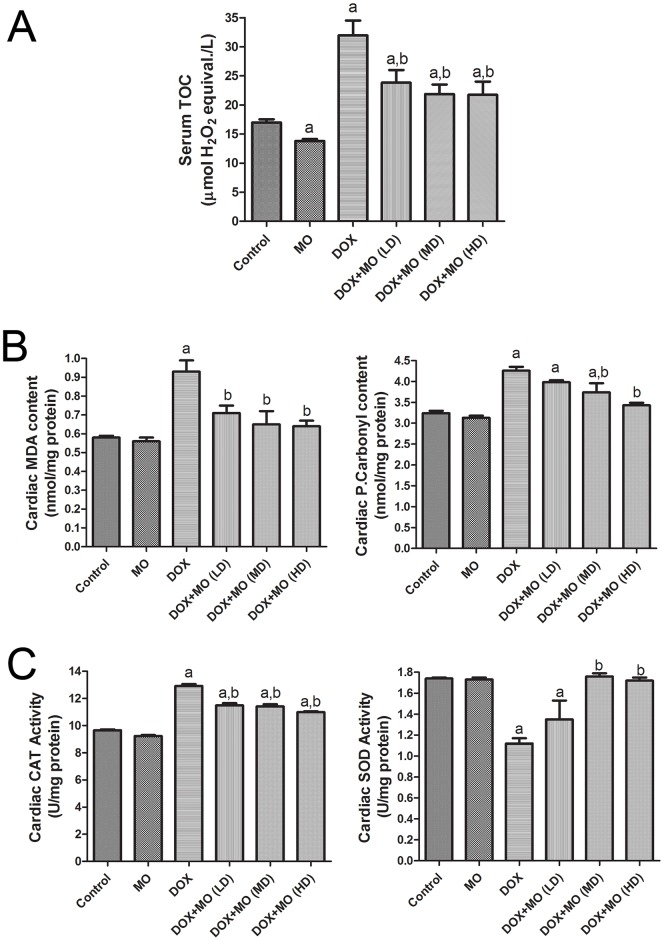
Effect of MO on oxidative stress biomarkers, (A) serum TOC and (B) cardiac MDA and P.Carbonyl levels and (C) antioxidant enzyme activities in DOX-treated rats. Data are represented as mean ± S.E.M. of seven independent rats of each group. a P<0.05 vs. control; b P<0.05 vs. DOX.

Compared to control group significant elevation in serum TOC and cardiac levels of MDA and P.carbonyl was shown in DOX-intoxicated group (Fig 2A and 2B). The concurrent treatment with MO and DOX significantly attenuated the elevations of these oxidative stress biomarkers. This effect was dose dependent where higher doses of MO extract abolished DOX-induced oxidative stress evidently than the low dose. MO alone had no effect on TOC and MDA and P.carbonyl contents compared to the control group. Cardiac tissues of DOX-treated rats showed significant depletion in SOD activity (Fig 2C). The activity of CAT enzyme was, however, elevated significantly in heart tissues of that group. Nevertheless, treatment with medium and high doses of MO restored SOD activities in protected groups compared to DOX-treated one. No significant changes in the activities of either CAT or SOD were observed in rats treated with MO alone compared to control.

#### Effect of MO on DOX-induced up regulation of inflammatory markers in heart

**Fig 3:**
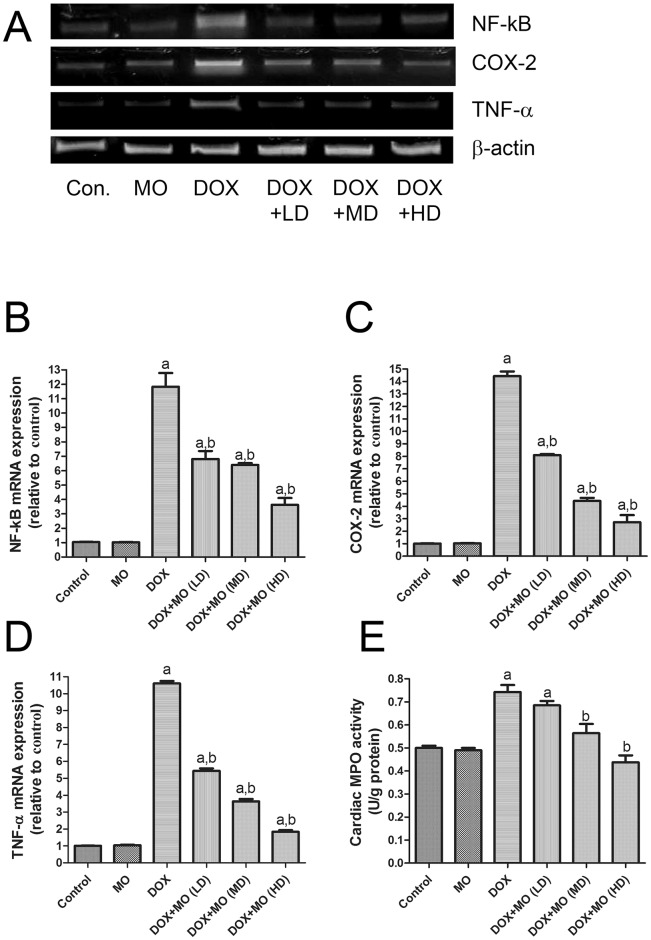
Effect of MO on inflammation related parameters. (A) Gel photograph depicting representative analysis of NF-κB, COX-2 and TNF-α mRNA expression. Data are expressed as fold change (relative to control group) and as mean ± S.E.M. of three independent rats of each group. (E) Cardiac MPO activity. Data represent as mean ± S.E.M. of seven independent rats of each group. a P<0.05 vs. control; b P<0.05 vs. DOX.

PCR and MPO assays were employed to analyze expressions of inflammatory markers in heart tissues of animals in different groups. Messenger-RNA levels of NF-kB, COX-2 and TNF-α as well as the activity of MPO are presented in fig 3. Our findings revealed that mRNA levels of NF-kB, COX-2 and TNF-α were significantly increased in DOX-treated rats compared to the control group. However, expression levels of all those transcripts were significantly decreased in the MO protected animal groups compared with the DOX group (Fig 3). Similarly, MPO activity was significantly elevated in heart tissues of DOX-treated rats compared to control values (Fig 3E). MO concurrent treatment with DOX significantly attenuated the elevations of such inflammatory markers in a dose-dependent manner.

#### Effect of MO on DOX-induced apoptosis in heart

**Fig 4:**
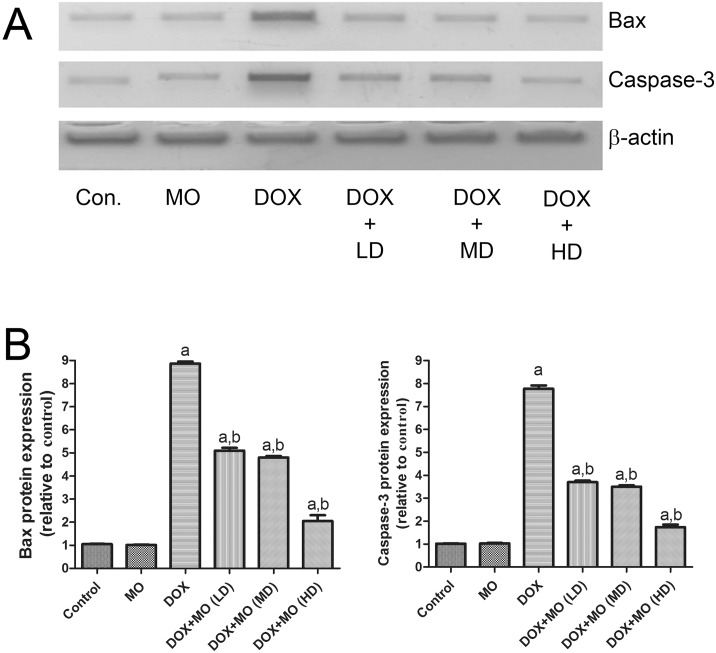
Effect of MO on cardiac apoptotic effect of DOX-treated rats. (A) Gel photograph depicting western blot analysis of bax and caspase-3 protein expression in heart of experimental animals. (B) Graphs present the relative expression of bax and caspase-3. Data are expressed as fold change (relative to control group) and as mean ± S.E.M. of seven independent rats of each group. a P<0.05 vs. control; b P<0.05 vs. DOX.

Protein expression of programed cell death (apoptosis) markers such as Bax and caspase- 3 were assessed in all tested groups using Western Blotting. Protein levels of Bax and caspase- 3 were significantly increased in DOX-treated rats compared to control. The DOX-induced upregulated expression of Bax and caspase- 3 proteins was significantly reduced in a dose-dependent manner by pretreatment with MO. Levels of protein expression of Bax and caspase-3 were not altered in animal group treated with MO alone, compared to the control group (Fig 4).

### Effects of MO, DOX and combined on cell toxicity and apoptosis in MCF-7

**Fig 5:**
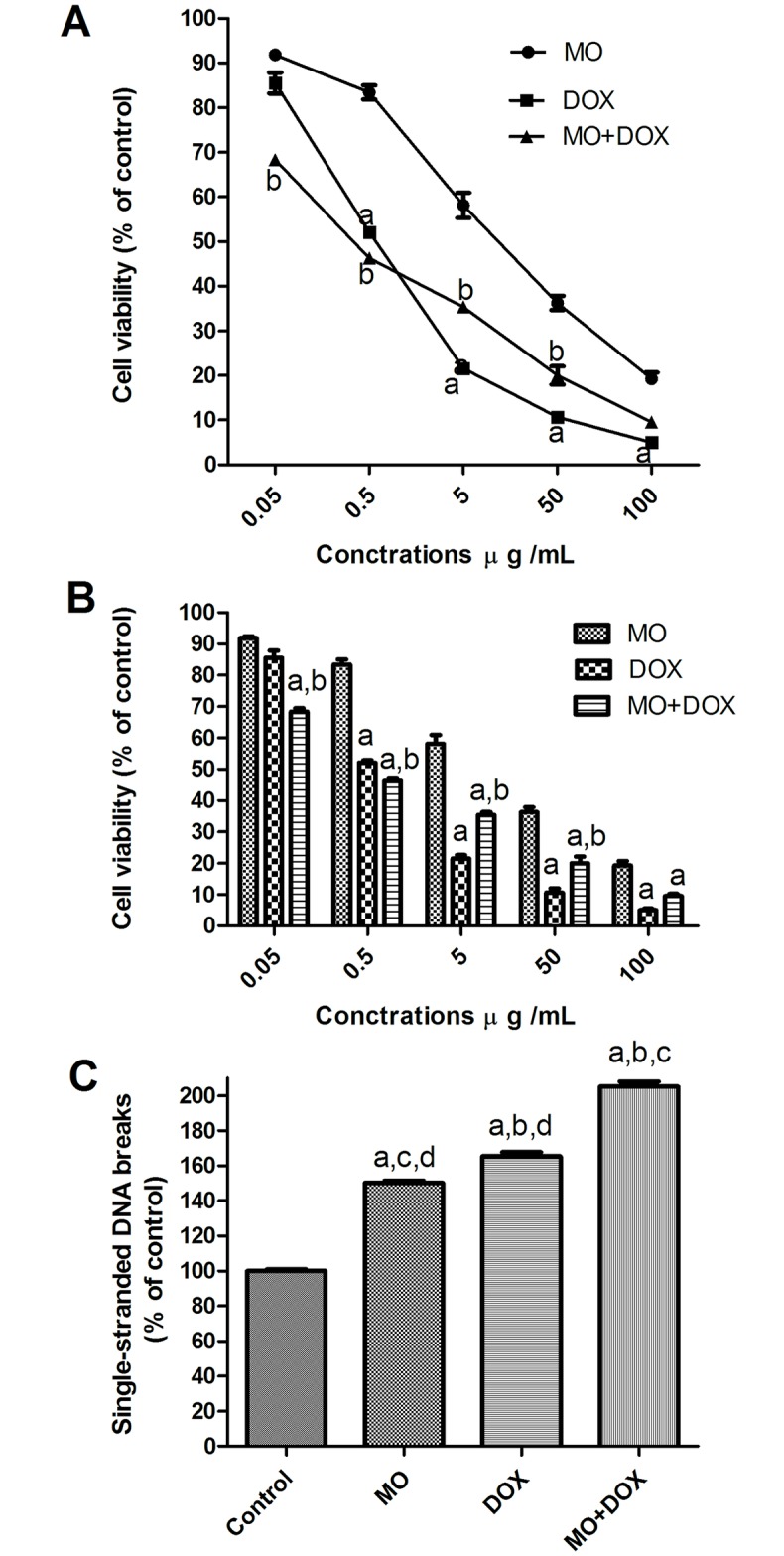
Effect of MO, DOX and their combination on viability and apoptosis of MCF-7 cells. (A and B) the effect of MO on the dose-response curve of DOX in MCF-7 cells. Cells were exposed to serial dilution of MO, DOX or combination of MO with DOX for 72h. Cell viability was after 72 h was measured using MTT assay. (C) Quantification of apoptosis induced by MO, DOX and their combination. The cells were treated with IC50 for 48 hrs. a P<0.05 vs. MO; b P<0.05 vs. DOX. Apoptosis was quantified by ELISA as single-stranded DNA. The data are expressed as percentages of control cells. Each point represents the mean ± S.E.M. of three independent experiments. a P<0.05 vs. control; b P<0.05 vs. MO; c P<0.05 vs. DOX; d P<0.05 vs. MO+DOX.

Effects of MO, DOX or both combined were investigated in human breast cancer cell line, MCF-7. A dose-dependent reduction in cell viability was recorded in cell treated with MO or DOX treatment (Fig 5A and 5B). The IC_50_ value of DOX was 0.818 μ g/mL (Table 3). Treatment with MO, as a single agent, significantly enhanced the mortality of cancer cells; however, MO was less potent than DOX with IC_50_ of 21.72 μ g/mL. The combination of MO with DOX significantly increased cell mortality compared with single DOX treatment, decreasing IC_50_ to half its original value (0.818 μ g/mL and 0.425 μ g/mL, respectively).

**Table 3:** IC_50_ of MO and DOX and their combinations on breast cancer cell line.

Treatment	IC_50_ μg/mL
MO	21.7 ± 0.12^b^
DOX	0.818 ± 0.01^a^
MO+DOX	0.425 ± 0.02^a^

IC_50_, concentration of drug needed to inhibit cell viability by 50%.

Data are presented as mean ± S.E.M. of three triplicate experiments. Significance was determined by ANOVA followed by Dunnett's t test:

^a^ P<0.05 vs. MO;

^b^ P<0.05, vs. MO+DOX.

Further, to assess whether the loss of cell viability by MO and DOX could, in part, be due to cell death, we evaluated apoptosis. APO Strand^™^ ELISA Apoptosis Assay was used, where single-stranded DNA breaks were indicative of apoptosis. APO Percentage^™^ assay demonstrated that the growth inhibitory effects of the MO, and DOX were associated with induction of apoptosis (Fig 5C). MCF-7 cells were treated for 48 hrs with MO (at 21.72 μ g/mL) or DOX (at 0.818 μ g/mL). The values for apoptosis 150 ± 1.3 and 165.4 ± 2.4 were obtained for MO and DOX-treated cells respectively compared to control-treated cells (0.1% DMSO). The most effective induction of apoptosis was seen in combination treatment with DOX+MO (205% ± 2.7).

#### Effects of MO and DOX on pro-apoptotic proteins in MCF-7 cells

**Fig 6:**
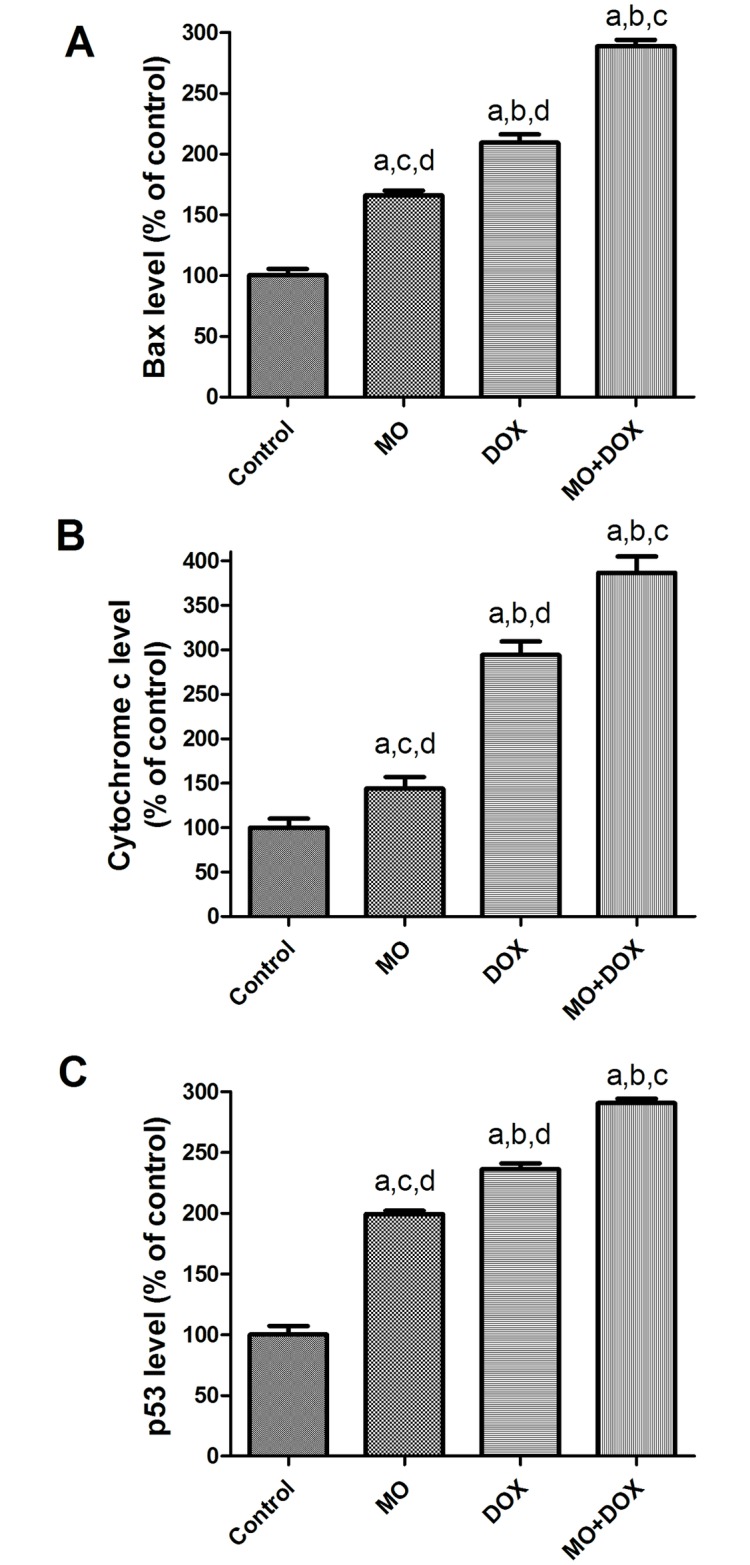
Effects of MO and DOX and their combination on pro-apoptotic proteins in MCF-7 cells. The protein expression of (A) Bax, (B) cytochrome c and (C) p53 levels were examined by ELISA in supernatant from MCF7 cells treated with IC50 of MO and DOX for 48 h. The data are expressed as percentages of control cells. Each point represents the mean ± S.E.M. of three independent experiments. a P<0.05 vs. control; b P<0.05 vs. MO; c P<0.05 vs. DOX; d P<0.05 vs. MO+DOX.

To determine the apoptotic signaling mechanisms responsible for the effects of MO and DOX and their combination, expressions of the pro-apoptotic proteins, Bax, cytochrome c and p53 levels were examined by ELISA, in supernatant of MCF-7 cells. As seen in fig 6A and 6B, levels of Bax and cytochrome c protein expressions were significantly up regulated in MO and DOX-treated cells, along with marked elevation of p53 levels, describing the role of the p53-mediated, mitochondrion-dependent, apoptotic pathway in MO and DOX on MCF-7 toxicity. Fig 6 showed that, MO at IC_50_ dose after 48 h significantly increased the level of Bax protein expression (165.87% vs control, DMSO- treated cells). The MO-induced Bax protein expression was significantly lower than that for DOX (209.28% vs control, DMSO- treated cells). Co-treatment of MO with DOX increased the level of protein expression (288.62% vs control, DMSO- treated cells) to a significantly higher level than that observed in DOX-treated cells. Similar to the changes in Bax protein expression, MO alone caused significant increase in the level of cytochrome c protein expression (143.74 of the control), while DOX determined significant increased the level of cytochrome c expression (294% of the control). Level of cytochrome c was even more increased by DOX and MO co-treatment (386.71 of the control). Similar alterations were observed in the level of p53 protein expression in cancer cells, MO significantly up-regulated the protein level of p53 (199.32% of the control), while DOX determined significant increased the levels of p53 protein expression (236% of the control). Marked increase of (290.48 of the control) was shown upon DOX/MO co-treatment. Induction of such apoptosis-related proteins in MCF-7 cells upon co-treatment with Dox and MO indicates a potent synergistic action of MO on DOX at multiple cellular levels of this pathway. It also depicts the function of the p53-mediated, mitochondrion-dependent, apoptotic pathway in MO and DOX–treated cells.

#### Effects of MO and DOX on intracellular redox state in MCF-7 cells

**Fig 7:**
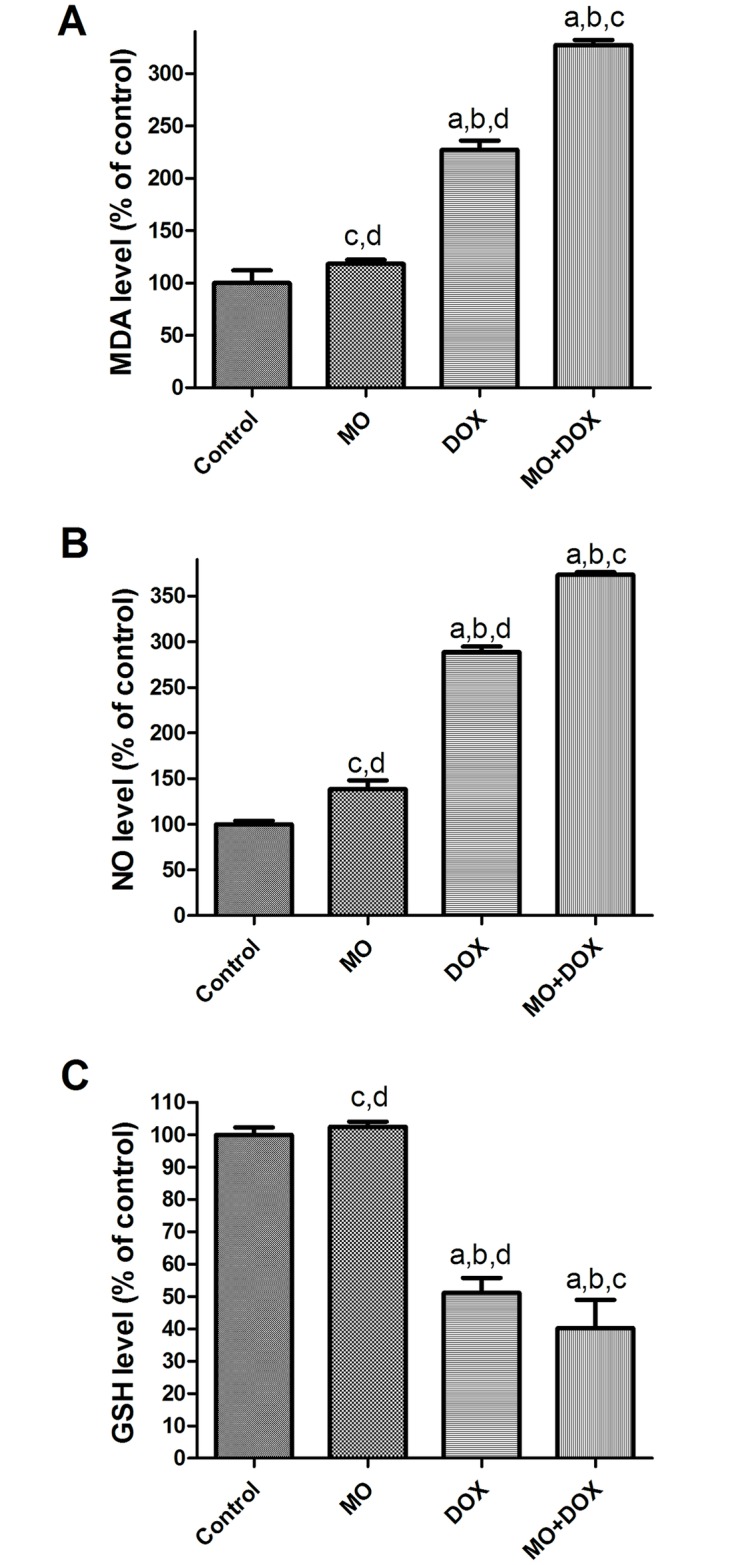
Effects of MO and DOX and their combination on intracellular redox state in MCF-7 cells. (A) MDA, (B) NO and (C) GSH levels were examined in supernatant from MCF7 cells treated with IC50 of MO and DOX for 48 h. The data are expressed as percentages of control cells. Each point represents the mean ± S.E.M. of three independent experiments. a P<0.05 vs. control; b P<0.05 vs. MO; c P<0.05 vs. DOX; d P<0.05 vs. MO+DOX.

To gain insights into the role of ROS formation during MO and DOX treatments or their combination in the mechanism of cell cytotoxicity in cancer cells, we measured the levels of intracellular oxidative stress-related markers (MDA, NO and GSH). As seen in fig 7A and 7B, levels of MDA and NO were significantly increased in cells given DOX, along with marked depletion of GSH levels, depicting the role of the oxidative stress in cell toxicity. The combined treatment with these agents was found to significantly increased MDA and NO levels and decreased in GSH level compared with either MO or DOX alone indicating the potent synergistic action of MO on DOX at nearly all cellular levels of these markers.

### Antioxidant activity of MO

**Fig 8:**
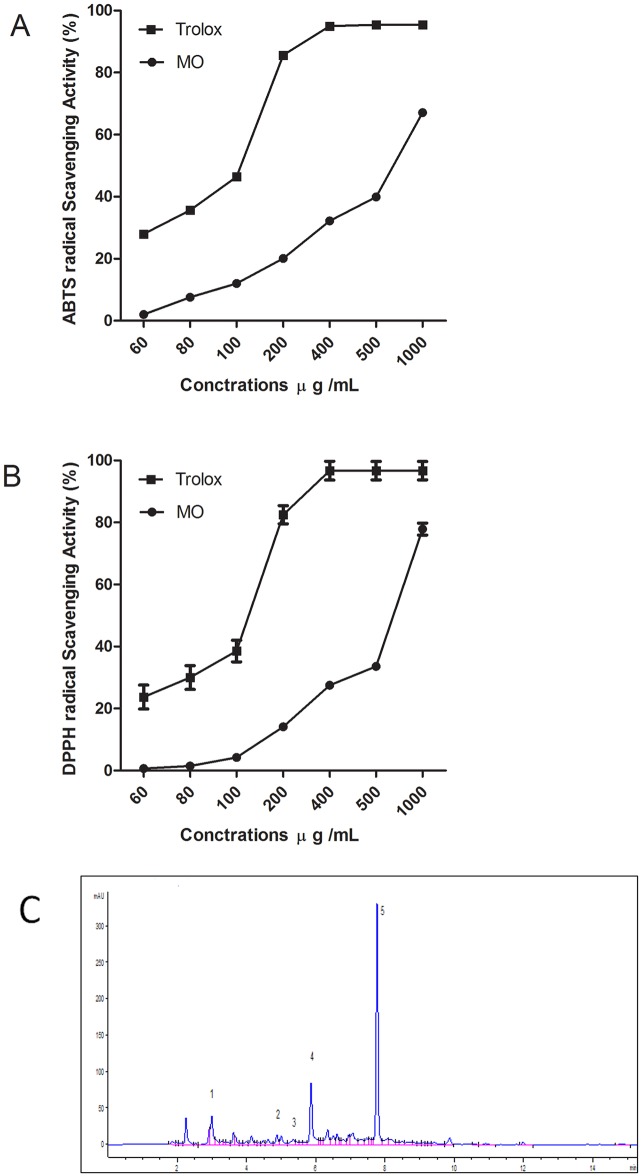
**(A)** ABTS, and **(B)** DPPH radical scavenging activities of MO ethanol extract and Trolox as reference antioxidant at various concentrations. **(C)** Representative HPLC phenolic acid profile of MO ethanol extract at 254 nm. Gallic acid (peak 1), caffeic acid (peak2), syringic acid (peak3), ferulic acid (peak4) and rosmarinic acid (peak 5). Values are means ± S.E.M. of three experiments.

In the present investigation, the FRAP, ABTS and DPPH• were used to determine the antioxidant activities of MO extracts. The results of the three assays are summarized in Table 4. The FRAP assay is based on the reduction of oxidized ferric ions to ferrous ions by a given antioxidant. The reducing capacity of a given compound serves as a reliable indicator of its antioxidant potential. In this study, each gram of dried MO has high FRAP value, 0.48 mmol ascorbic acid equivalent. ABTS are other radical- scavenging methods that are broadly used to assess the ability of natural extracts to scavenge free radicals generated from those reagents. The MO exhibited high anti free radical scavenging activity where the ascorbic acid equivalent antioxidant capacities of the MO were 0.79±0.08 and 0.73±0.02 mmol/g in ABTS and DPPH assays, respectively. The ABTS and DPPH radical scavenging ability of samples (IC_50_) was 677.7± 0.39 and 743.6±1.29 μg/mL. The effect of MO was dose dependent (Fig 8A and 8B).

**Table 4:** Total antioxidant activity of ethanol extract from MO expressed as ascorbic acid equivalents (mmol/g of dry extract) and as IC_50_ (μg/mL of dry extract). Trolox was used as positive control.

Extract	FRAP Assay	ABTS Assay		DPPH Assay	
	TAC mmol /g	TAC (mmol/g)	IC_50_ μg/mL	TAC (mmol/g)	IC_50_ μg/mL
MO	0.48±0.01	0.79±0.08	677.7± 0.39	0.73±0.02	743.6±1.29
Trolox	3.49±0.09	3.64±0.50	107.6± 0.30	3.62±0.05	129.9± 0.05

Values are means ± SME of three triplicate experiments.

#### The amount of total phenolics, flavonoids and HPLC quantification of major phytochemical compounds

The Folin–Ciocalteau’s assay was employed to estimate the total phenolics and flavonoids of various plant extracts while the aluminum chloride method was used to determine the total flavonoids. The overall phenolic and flavonoid contents of examined extract were determined and expressed in milligrams (mg) of gallic acid and catechin equivalents, respectively (Table 4). As shown in Table 5, the plant extract has 192.4 ± 2.33 mg gallic acid and 74.1 ± 1.13 mg quercetin, as total phenolic and flavonoid contents, respectively.

**Table 5:** Amount of total phenolics^a^, flavonoids^b^ and the qualitative–quantitative analysis^c^ of the ethanol extract from MO carried out using an HPLC-DAD.

Compound	Amount of compounds	%
Total phenolic content	192.400 ± 2.33	19.24%
Total flavonoid content	74.100 ± 1.13	7.41%
Gallic acid	0.223 ± 0.03	0.02%
Caffeic acid	0.198 ± 0.09	0.02%
Syringic acid	0.112 ± 0.05	0.01%
Ferulic acid	1.585 ± 0.09	0.16%
Rosmarinic acid	75.320 ± 1.13	7.53%
Oleanolic acid	0.866 ± 0.13	0.09%
Urosolic acid	1.403 ± 0.10	0.14%

Results are expressed as mean ± SEM of three experiments.

^a^ Total phenolic content was expressed as mg gallic acid equivalents/g dried extract.

^b^ Total flavonoid content was expressed as mg catechin equivalents/g dried extract.

^c^ The amount of compounds was expressed as mg/g of dried extract.

Data collected from qualitative–quantitative analysis of MO extract utilizing HPLC coupled with diode array detection (DAD), is presented in Table 5. The indicator wavelengths of 280, 254 and 320 nm were selected to compare the number of peaks and their separation. At wavelength of 254 nm the HPLC chromatograms showed more peaks than those revealed at 280 and 320 nm. Therefore, the wavelength of 254 nm was selected for further separation and quantitation of the phenolic acids examined here. The components caffeic acid, syringic acid acid, gallic acid, rosmarinic acid, and ferulic acid (Fig 8C) were characterized by comparisons to the standard counterparts’ retention times and UV spectra analyzed under identical analytical conditions. The quantitative data was, however, calculated from their respective calibration curves. The plant extract’s major component was identified as rosmarinic acid (75.32 mg/g) that represented 7.532% of the extract. On the other hand, the least abundant compounds present in the extract under investigation was syringic acid (0.198 mg/g) that represented 0.02% of the extract. The other identified triterpene acids were oleanolic acid (0.866 mg/ g) and urosolic acid (1.403 mg/ g) and phenolic compound (Table 5).

## Discussion

Despite the notable advancement in targeted therapies in some cancers; major hurdles remains. Most targeted therapies are highly toxic and patients often experience relapse after a brief disease-free intervals. Tumors’ genetic heterogeneity accounts for such relapses in most cases. Low-toxicity phytochemicals could support conventional therapeutic approach by targeting key pathways and mechanisms [35].

In this study, we showed the effect of MO in ameliorating DOX-induced acute cardiotoxicity in rats and in potentiating the efficacy of DOX against breast cancer cells. DOX-induced cardiotoxicity was evaluated biochemically through assessing levels of AST, CK and CK-MB and histopathologically via examining integrity of the heart tissues. DOX-intoxicated group showed significant activity boost of serum AST, CK and CK-MB. Those are conventional biomarkers that are reported to be released from damaged myocytes in association with cardiotoxicity [36]. DOX-induced myocardial injury was further confirmed histopathologically where myocyte necrosis, cytoplasmic vacuolization, interstitial edema and hemorrhage degeneration, and inflammatory cells infiltrations were clearly shown. Similar histopathological and biochemical marker alterations have been previously reported in acute DOX-induced cardiotoxicity [4; 7; 19; 37]. MO pretreatment, on the other hand, significantly ameliorated DOX changes and inhibited elevations of serum tested enzymes as well as, almost restoring the normal architecture of the heart. DOX- associated abnormalities have been gradually abolished with the various applied doses of MO. Optimal effect was reached in animals treated with 750 mg/kg MO, suggesting a protective role of MO against DOX cardiotoxicity.

We, then, investigated the possible molecular mechanisms underlying the cardio protective effects of MO. Oxidative stress has been reported among the main contributing factors to the DOX-induced deformation of heart tissues [2; 4]. The ring structure of anthracycline of DOX has been shown to increase both enzymatic and nonenzymatic single-electron redox cycle liberation of ROS from molecular oxygen [38]. Different studies have shown that free radicals deplete the antioxidant defense system and consequently boost the oxidation process of both lipids and proteins in heart tissues of DOX-treated rats [2; 4; 39]. Free radical scavenging, thus, provides important ways to protect against DOX-induced oxidative injury. In the present study, DOX-induced oxidative stress was manifested through the elevation of oxidized lipids (MDA) and proteins (P. carbonyl) in heart tissues and the serum TOC. Interestingly, the DOX-induced oxidation of lipids and proteins was prevented by MO in dose-dependent manner, suggesting an antioxidant effect of this herb.

Multiple endogenous antioxidant enzymes including, but not limited to, SOD and CAT normally represent the first line of cell defense against oxidative stress-mediated cardiac injury. Those enzymes work in concert to detoxify superoxide radicals and hydrogen peroxide in cells [40]. DOX reduced the antioxidant activity of SOD enzyme in the heart and this depletion in antioxidant defense mechanism was associated with the elevation of CAT activity, an enzyme that eliminatesH_2_O_2_ [40], in the heart and other tissues. The present results indicated that pretreatment of MO caused an increase in the activity of SOD activity and decrease in CAT activity in DOX–treated rats. Similar results have been shown in rodents treated with an acute dose of DOX [7; 39]. This explains, at least in part, the massive production of H_2_O_2_ and its significant role in DOX-induced cardiotoxicity. It has been reported that once cytochrome P450 reductase and NADPH was present, the redox-cycle of DOX was a major source of H_2_O_2_ in tissues [38].

The MO antioxidant capacity was determined by assessing its scavenging activity on ABTS and DPPH radicals and its reducing ability by FRAP assay. The present results showed that the MO exhibited significantly higher ABTS and DPPH scavenging activities and demonstrated its potent hydrogen-donating ability as well [41]. Polyphenol compounds are known for their potent redox properties. Such a property allows those compounds to effectively adsorb and neutralize free radicals, quench singlet and triplet oxygen, or decompose peroxides [41]. We show here a good correlation between the antioxidant activity and the phenolic and flavonoid contents in of MO extract. Therefore, phenolic compounds seem to be responsible for the antioxidant activity of MO extract. These results insinuated that the cardioprotective effect of MO against DOX cardiotoxicity might largely be attributable to its evident antioxidant capacity.

Among many other deleterious effects, oxidative stress may cause inflammation through activating redox sensitive transcription factors, such as NF-κB [42]. NF-κB works as a link between oxidative-induced damage and inflammation. It also increases the expression of a battery of distinct pro-inflammatory mediators such as TNF-α, COX-2and iNOS [42]. Several studies have reported that DOX induces a series of inflammatory reaction in heart tissues by up regulating NF-κB and stimulating subsequent pro-inflammatory cytokines production [19; 37]. In the present study, DOX intoxication significantly up regulated NF-κB, TNF-α and COX-2 gene expressions concurrent with elevation cardiac MPO activity reflecting an amplified inflammatory responses. On the contrary, MO pretreatment significantly down regulated the expression of NF-κB and hence inhibited the downstream inflammatory cascade as evidenced by decreasing the expression of TNF-α and COX-2 and the levels of MPO, so MO provides an evident anti-inflammatory effect.

Oxidative stress has been reported to elicit mitochondrial-dependent (intrinsic) apoptotic pathway [43]. Induced level of ROS up regulates Bax expression leading to permeabilizing the external mitochondrial membrane, the release of cytochrome c and the activation of caspases which ultimately lead to the apoptotic degradation phase [44]. In agreement with previous study, we showed here that DOX intoxication induced significant increase in Bax and caspase-3 protein expressions [19]. Furthermore, DOX intoxication induce increase in Bax, cytochrome c and P53 protein levels in breast cancer cell line, MCF-7, which was in agreement with the previous *in vitro* study [45]. The apoptotic effect to DOX could be attributed to triggering the intrinsic mitochondrial-dependent apoptosis pathway through the generation of ROS as shown by the induced oxidative stress observed in the DOX-treated rats. Pretreatment with MO resulted in significant decrease in Bax and caspase-3 protein expressions. This anti-apoptotic effect of MO can be attributed to a free radical scavenging capability.

Consistent with the action of MO on MCF-7 reported previously [14], the present *in vitro* analysis indicates that MO may boost DOX-induced elevation of protein levels of pro-apoptotic Bax, cytochrome c and p53 in human breast cancer MCF-7 cell line. Reportedly, those pro-apoptotic proteins are believed to bind to the mitochondria and thus regulating apoptosis through modulation of the mitochondria permeability [46]. Interestingly, besides the p53-dependent transactivation of apoptotic genes, this tumor suppressor protein may also directly bind to and suppress the Bcl2 proteins, leading to the release of cytochrome C and the instigation of caspase cascade [47].

Furthermore, we demonstrated for the first time that induced levels of ROS were necessary for the apoptotic effects of MO. The apoptotic effect of MO, DOX and their combinations was mediated by inducing oxidative stress as well as GSH depletion in cancer cells. We show here that MO exerted pro-oxidative rather than anti-oxidative effects due to ROS formation in treated breast cancer cells. This result provides evidence for the anticancer activity of the studied plant on specific cell line and suggests that cell-killing property of MO could be mediated by ROS, thus involving mechanisms independent of the plant’s free radical scavenging activities. This preferential cytotoxicity of plant polyphenols against cancer cells is explained by the observation made by [47,48], where authors showed that copper levels in cancer cells were significantly elevated and confirmed that mobilization of endogenous copper and then ROS production by polyphenols was critical for the triggering of pro-oxidant cell death. Further in vivo analyses will soon be underway to extend our understanding of the mechanisms described in vitro in the present study.

Thanks to the phenols capacity as hydrogen- or electron-donating agents, and to their unique properties as metal ion chelating compounds, phenols demonstrate substantial free radical scavenging activities [41]. Flavonoids are a class of secondary plant phenolic with powerful antioxidant and cardio protective properties [41]. The high content of total phenols and flavonoids reported here for the ethanolic leaves extract of MO is in line with previous studies on this plant [13; 48]. Lin et al. [13] reported that for MO cultivated in Taiwan, the ethanolic extract of its leaves contained phenolic acids including gallic, caffeic, p-coumaric, protocatechuic, chlorogenic, and rosmarinic acid, as well as flavonoids including (+) p-catechin, p-epicatechin, hesperetin, eriodictiol, hesperidin, naringin, lutenolin, and naringenin where rosmarinic acid was the major ingredient. In the present investigation, the detected phenolic compounds in our MO samples were gallic, caffeic, syringic, ferulic acid, and rosmarinic acid. Rosmarinic acid was also the main compound of the plant extract which is consistent with earlier studies [13; 15; 49]. The average content of phenolic acids analyzed here in the present lemon balm samples is quite comparable with contents reported elsewhere [15]. Finally, and in agreement with previous study [10], well known antioxidants like triterpene acids, oleanolic acid and ursolic acid were also detected in our MO samples. Therefore, phenolic acids and triterpene acids in the present extract may contributes synergistically to the beneficial properties of MO as antioxidant *in vivo* and pro-oxidants in vitro.

## Conclusions

**Fig 9:**
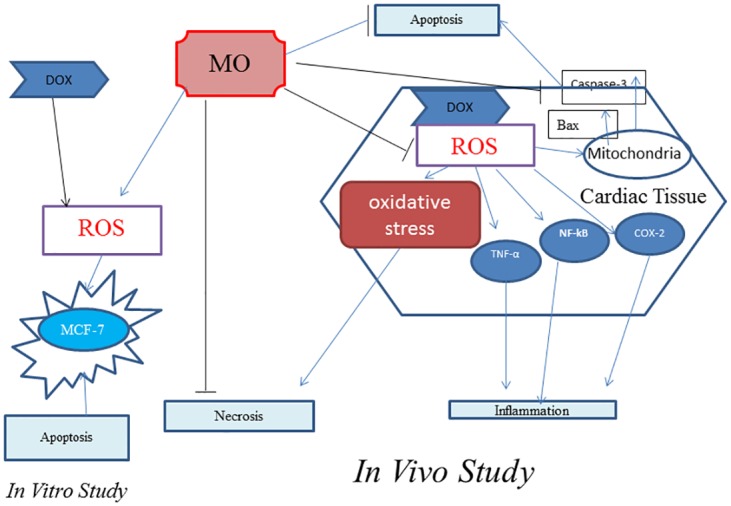
MO blocks DOX-induced apoptosis, -ROS formation and –necrosis and down regulates the induced inflammation *in vivo*. In human breast cancer cells (MCF-7), MO improves the anticancer efficacy of DOX and potentiates oxidative damage and apoptosis.

The present study substantiates the promising ameliorating effects of MO against DOX-induced cardiotoxicity in rats through modulation of oxidative stress, diminution of inflammation and abrogation of apoptosis in rat heart (Fig 9). Identification of a mechanism for MO anticancer effect introduces the possibility that combining this plant with DOX might enhance the therapeutic efficacy of DOX in clinical oncology. Beneficial effect of the MO extract is likely due to the synergistic interactions of phenolic compounds and other triterpene acids of MO. Accordingly, this study provides new insights into the development of strategies to augment anticancer activity of DOX and further to alleviate its cardiotoxicity. Further confirmatory studies both at preclinical and clinical levels are needs to evaluate a combination therapy.

It is important to point out here that while the current study used an acute model of DOX cardiotoxicity rather than chronic DOX administration as occurs clinically, the results provide proof of concept that MO may be beneficial for DOX-induced cardiotoxicity. This, however, needs to be confirmed in a more clinically relevant model to explicate the mechanism and develop strategies in prevention against DOX-induced cardiotoxicity.

## Abbreviations

ABTS: 2,2-azino-bis(3- ethylbenzothiazoline-6-sulfonate)
AST: Aspartate aminotransferase
MDA: malondialdehyde
CAT: catalase
CK: Creatine kinase
CK-MB: CK isoenzyme-MB
COX-2: Cyclooxygenase-2
DOX: Doxorubicin
DPPH: 1,1-diphenyl-2-picrylhydrazyl
MPO: Myeloperoxidase
NF-kB: nuclear factor-kappa B
NO: nitric oxide
P.carbonyl: protein carbonyl
SOD: superoxide dismutase
TNF-α: Tumor necrosis alpha
